# Endoscopic endonasal transclival approach for resection of a pontine cavernous malformation

**DOI:** 10.3171/2019.7.FocusVid.19212

**Published:** 2019-07-01

**Authors:** Lucas Ramos Lima, Jarbas Carvalhais Reis, Gerival Vieira Junior, Tiago Fraga Vieira, Lucidio Duarte de Souza Filho, Tiago Silva e Carvalho, Fabricio Nery Marques, Ramon Souza Lago, Thiago Vinícius Muniz Santana

**Affiliations:** Departments of 1 Neurosurgery and; 2 Otolaryngology, Hospital São Francisco de Assis;; 3 Department of Neurosurgery, Biocor Instituto;; 4 Department of Neurosurgery, Hospital Felicio Rocho; and; 5 Department of Neurosurgery, Hospital Vila da Serra, Belo Horizonte, Minas Gerais, Brazil

**Keywords:** brainstem, cavernous malformation, endonasal, endoscopic, transclival, video

## Abstract

Symptomatic cavernous malformations in the ventral region of the pons are difficult to access surgically. The authors present a case of a 46-year-old woman with a 10-year history of sudden and transitory diplopia and right hemiparesis, followed by five more episodes of mild right hemiparesis. Brain MRI showed a 2.6-cm cavernous malformation in the pons with an exophytic portion in the prepontine cistern. The patient underwent an endoscopic endonasal transclival approach for a complete resection of the lesion. CSF leak was noted and corrected on the sixth postoperative day. The patient progressed with complete motor deficit recovery.

The video can be found here: https://youtu.be/ePgpyij2Wpo.

## Transcript

This video demonstrates a complete resection of a pontine cavernous malformation through an endoscopic endonasal transclival approach.

A 46-year-old lady presented sudden diplopia and right hemiparesis 10 years ago with complete neurological recovery, followed by five more episodes of mild right hemiparesis. The last episode occurred 6 months before the surgical procedure. The MRI suggested a cavernous malformation in the pons with an exophytic portion in the prepontine cistern, and the decision was made to take the patient to the operating room for resection of the lesion through an endoscopic endonasal transclival approach.

The patient was positioned supine with the head slightly turned to the right side and tilted to the left, fixed in a Mayfield head pins.

The middle and superior turbinates were resected bilaterally. A nasoseptal flap was made in the left nostril and positioned in the nasopharynx. A reverse flap was made on the right side.

After extensive opening of the sphenoid sinus, we drilled the septations to expose the middle clivus between the paraclival carotid arteries, below the sella. The middle clivus was removed to expose the dura of the posterior fossa.

We planned a left paramedian incision to avoid the basilar artery in the midline and the abducent nerve laterally. Doppler ultrasound and direct electrical stimulation were used. Bleeding from the basilar plexus was controlled with gelatin hemostatic matrix. A vertical incision was made, and we visualized the exophytic portion of the cavernous malformation in contact with the basilar artery. The cavernous malformation was carefully dissected from the basilar artery and its branches.

The incision of the dura was enlarged laterally and the resection of the lesion was started in piecemeal. Microsurgical techniques were adopted, in the same way they would be used in traditional microsurgical approaches. The peripheral portions of the lesion were pulled into the center of the surgical cavity. Most of the time, resection was performed by traction with a grasping forceps and countertraction with a suction tube.

The pontine tissue with hemosiderin was maintained on surgical borders. Careful dissection of pontine arterial branches was performed. Removal of the lesion proceeded in piecemeal. A large clot in the lateral portion of the lesion was mobilized and removed. The last piece of the lesion was removed and the pontine parenchyma with hemosiderin was inspected.

The surgical cavity was covered with oxidized cellulose hemostatic agent. The reconstruction was performed with an inlay and an onlay fascia lata, followed by fat, and the positioning of the nasoseptal flap. A Foley catheter was used to maintain the position of the nasoseptal flap.

There was a slight worsening of right hemiparesis in the postoperative period. The patient presented CSF leak on the sixth postoperative day, which was corrected the same day with a surgical revision. We noticed that the pulsation of the basilar artery displaced the layers of fascia lata. These layers were repositioned and fibrin glue was used to prevent further displacement. Fat and the nasoseptal flap were repositioned, followed by the Foley catheter. A lumbar drain was implanted.

An MRI performed after 6 months of surgery showed cicatricial alterations in the surgical borders with contrast enhancement, but no obvious residual lesion. There was complete resolution of the motor deficit.

We decided to propose surgical treatment, since the patient presented multiple episodes of bleeding, and a new episode could be catastrophic. Traditional approaches were considered. Retrosigmoid craniotomies, anterior petrosectomies, and presigmoid approaches are some options. All of them expose the lateral portion of the pons and allow access through safe entry zones, such as the peritrigeminal zone and the middle cerebellar peduncle zone. However, manipulation through the pontine parenchyma is required, and there is a risk of injury to eloquent areas, especially because there is important displacement of the normal structures, making anatomical recognition difficult. Also, the surgical corridor is narrow, in a lateral route between the cranial nerves.

We opted for an endoscopic endonasal transclival approach since it provides direct access to the ventral region of the pons and would allow us to access the lesion without having to cross the pontine parenchyma. The biggest disadvantage of this approach is the CSF leak rate, which is still higher than in traditional approaches. However, the advancement of surgical techniques has reduced its rate, and even when it occurs, it is most often treated with revision of the reconstruction.

We have shown the resection of a cavernous malformation in the ventral pons with a good functional outcome, and we believe that this case may reinforce the idea that the endonasal transclival approach is a good option for selected cases.

### Time points

0:30 Patient’s history
1:05 Positioning
1:15 Nasal stage
1:47 Sphenoid sinus opening
2:05 Relevant anatomy
2:26 Dura mater opening
3:01 Cavernous malformation dissection
3:19 Piecemial removal
5:26 Surgical cavity inspection
5:42 Reconstruction
5:57 Follow-up
6:31 Postoperative images
6:53 Discussion
8:19 Conclusion
